# Exposure assessment of wastewater treatment plant employees to BTEX: a biological monitoring approach

**DOI:** 10.1038/s41598-022-25876-x

**Published:** 2022-12-12

**Authors:** Mansooreh Dehghani, Alireza Abbasi, Ziba Taherzadeh, Samaneh Dehghani

**Affiliations:** 1 Research Center for Health Sciences, Institute of Health, Shiraz University of Medical Sciences, Shiraz, Iran; 2 Department of Environmental Health Engineering, School of Health, Shiraz University of Medical Sciences, Shiraz, Iran; 3 Department of Occupational Health Engineering, School of Health, Shiraz University of Medical Sciences, Shiraz, Iran; 4 Department of Environmental Health Engineering, School of Public Health, Tehran University of Medical Sciences, Tehran, Iran

**Keywords:** Environmental sciences, Risk factors

## Abstract

To monitor employees' work safety and exposure against air contaminants, Trans, trans-muconic acid, Hippuric acid, Methyl hippuric acid, Mandelic acid and Phenylglyoxylic acid can be used as reliable biomarkers of exposure to benzene, toluene, ethylbenzene, and xylene (BTEX) compounds. This study aims to determine the level of urinary metabolites of BTEX compounds using biological monitoring in the employees of a wastewater treatment plant (WWTP) in the south of Iran. The study was performed on 56 employees of the WWTP of one of the southern cities of Iran in 2020. Urine samples (n total = 112) consisting of 60 samples of employees working in the operation section (exposed group) and 52 samples of employees working in the administrative section (control group) in the WWTP were collected before and at the end of their shift. The mean concentration of urinary metabolites of BTEX of both groups ranged from 546.43 (μg/g cr) for trans, trans-muconic acid to 0.006 (μg/g cr) for methyl hippuric acid, which indicates that most of the evaluated metabolites showed a higher concentration than their occupational threshold limit value urine (p < 0.05). Regression analysis results showed a significant correlation (p < 0.05) between age and utilization of flame heaters with changes in the measured BTEX metabolites in the urine. The results of this study illustrate that WWTPs should be considered as one of the workplaces with potential sources of BTEX exposure for employees. Future investigations are recommended to perform itemized appraisals of BTEX intake sources, particularly in employees of the operational sections of WWTP.

## Introduction

Benzene, toluene, ethylbenzene, and isomers of xylene (BTEX) are classified among the most important volatile compounds (VOCs) released into the environment from natural and manufactured resources^1^. Although traffic is a critical source of atmospheric BTEX emissions in cities^2^, workers in various occupations are exposed to BTEX compounds^3,4^. VOC emissions in wastewater treatment plants (WWTPs) depends on several agents, including the size of the wastewater treatment plant, the amount and characteristics of influent, the wastewater treatment processes and units, and the technologies applied to control or remove VOCs^5^. Like all of the VOCs, BTEX compounds are organic molecules with high vapor pressure and low boiling points^6^. The emission of these compounds may be because of the concentration gradient between the air and the water phase in WWTPs processes and turbulence in the aeration processes^7^.

The concentration of BTEX and the duration of exposure to them are among the most important factors affecting the health condition of people who are exposed to these compounds^8^. Although inhalation is a standard exposure method to BTEX compounds, dermal absorption should also be considered^9^. BTEX compounds have a wide range of neurological side effects, including weakness, loss of appetite, fatigue, confusion and nausea, respiratory complications, and hematologic health consequences, including cancer^10^. According to the IARC, benzene has long been considered a carcinogen^11^, and the ACGIH standard, the permissible threshold for 8-h exposure is 0.5 ppm^12^. Toluene, xylene, and ethylbenzene also have toxicological effects on the nervous system and are considered carcinogenic for humans^13–15^.

Occupational exposure to chemicals can be evaluated by determining environmental concentrations in different workplaces or byby determining environmental concentrations in various workplaces by calculating occupational exposures according to workers' work patterns (e.g., time spent in each workplace). Biomonitoring is another method, unlike environmental assessment of pollutants and chemicals, which assesses exposure from all ways and sources to determine the various contaminants^16^. Urine metabolites may act as indicators of occupational or environmental exposure, especially for water-soluble chemicalssuch as benzene, which includes phenol, hydroquinone, tt-MA acid, urinary hippuric acid (uHA), and S-phenylmercuric acid^17–19^. Among them, urinary tt-MA is recognized as a reliable and relatively facile biomarker^20^ and represents about 1% of the absorbed amount of benzene^21^. The range of metabolic conversion of benzene to tt-MA is about 2–25% and dependent on the benzene exposure level, simultaneous exposure to toluene, and probably also to genetic factors^22^. Recent studies have shown that trans,trans-muconic acid, a minor benzene metabolite, can be determined using HPLC with UV detection^23^.

Furthermore, tt-MA is known to be involved in bone marrow leukemogenesis, its applications in biological monitoring could thus be important in risk assessment^24^. Despite the low metabolic conversion of benzene to tt-MA, ACGIH recommends it as a suitable biomarker for occupational exposure assessment to benzene (higher than 0.25 ppm) in limited quantities (biological exposure index) of 500 μg/g cr^25^. Due to the short half-life of BTEX compounds in the human body^26^, assessing BTEX metabolized compounds in the urine can be more accurate in recognizing the rate of exposure^27^.

Recent occupational research using biomonitoring to assess workers' exposure, like in gas station workers^28^, petrochemical plants^29,30^, composting plant employees^31^, and traffic police personnel^32^, have been examined. Despite confirming the presence of BTEX compounds in the air of this wastewater treatment plant (WWTP) and as a result of potential exposure of the WWTPs employees to BTEX (Dehghani et al. 2021b, Dehghani et al. 2018), estimation of the biological levels of BTEX among the employees of WWTP has not been considered so far. We assessed WWTPs employees' exposure to BTEX compounds by measuring their urinary metabolites, including Trans, trans-muconic acid (tt-MA), Hippuric acid (HA), Methyl hippuric acid (MHA), Mandelic acid (MA), and Phenylglyoxylic acid (PGA) as a valuable biomarker to assess the personal exposure to benzene, toluene, ethylbenzene, and *m*+*p*-xylene, respectively. A questionnaire will obtain a systematic baseline among the participants, namely the control group (administrative personnel) and the exposed group (operational crew). The level of biomarkers among the participants before and after their shift allows immediate and accurate exposure estimation, and the post-hoc statistical analysis will determine the levels, significance, and importance for human health.

## Materials and methods

### Subject of study

This descriptive-analytical study was carried out in February 2020. The subjects of this study were 56 employees of the administrative and operational units of the wastewater treatment plant (WWTP) aged 25 to 59 years (Table 2, demographic characteristics). The spot sampling method (36) was selected so that the samples were representative of all employees of the WWTP (operational unit and administrative unit). The study was accompanied by a 20- to 30-min interview using a questionnaire that included demographic, job, and workplace information, condition of traffic near the living area, lifestyle, eating habits, smoking status, secondary exposure to smoke, and exposure history to BTEX. The studied WWTP is located in Iran. The mean discharge of the treatment plant is 930 L/s, and the peak discharge of transmission lines to the treatment plant is 2600 L/s. The length of transmission lines to the treatment plant is 5.5 km.

### Collection of biological samples

To carry out the study and biological monitoring of urinary metabolites of BTEX compounds, after obtaining consent and conducting interviews with the participants, 30 samples of operational employees (all the employees in the operational unit) in the WWTP, as the exposed group, and 26 random samples of the administrative employees were chosen, as a control group. In either group, one sample was taken before the start of the work shift (before the exposure), and the second sample was taken after the end of the shift (after the exposure); 112 urine samples were collected using special containers for urine sampling and were immediately wrapped in aluminum foil (to prevent the penetration of sunlight). All samples were transferred to the laboratory using a cool box and kept at − 20 °C until the analysis.

### Approval statement

The informed consent form was received from each subject. Besides, Shiraz University of Medical Sciences Ethics Committee approved the study protocol by the code “IR.SUMS.REC.1398.1267”. We confirm that all methods were performed following the relevant guidelines and regulations.

### Sample preparation and analysis

To measure the urinary metabolite trans, trans-muconic acid (ttMA), solid-phase extraction (SPE) was first performed using a potent anion exchange cartridge (SAX—60 mg/3 mL) with 3 mL of methanol and 3 mL. 1 mL of urine sample was used, and then the cartridge was washed with 3 mL of 1% aqueous acetic acid. The ttMA elution was performed using 3 mL of 10% aqueous acetic acid. For the chromatographic analysis, 20 μL of the sample was injected into HPLC (KNAUER, Azura,) with PDA UV–Vis Detector, equipped with a reverse-phase C18 column (Eurospher 100-5, 250 × 4 mm—KNAUER). The flow rate was set to 1 mL per minute and detected at 265 nm.

Extraction and analysis of urinary Hippuric acid (HA) and Methyl hippuric acid (MHA) metabolites were performed according to NIOSH 8301 standard method. Briefly, 80 μL of 6 N HCl was mixed with 1 mL of a urine sample, and then 0.3 g of NaCl was added. 200 μL of the organic layer of the resulting mixture was transferred to a microtube, evaporated, and dried under a stream of nitrogen gas. Then it was redissolved in 200 μl of the utilized mobile phase of HPLC. HPLC analysis was implemented using a C18 column with water/acetonitrile/glacial acetic acid (84:16:0.025% (v/v)) as the mobile phase with a 1 ml per minute flow rate. The column temperature was adjusted to 37 °C, and the detection wavelength was set at 254 nm.

Determination of Mandelic acid (MA) and Phenylglyoxylic acid (PGA) was also accomplished by liquid–liquid extraction based on the method previously reported in ethyl acetate^34^. Then HPLC analysis was performed using a reverse-phase C18 column (Eurospher 100-5, 250 × 4 mm—KNAUER) with a constant temperature of 25 °C. Water: Methanol 90:10 solution (v/v) containing 0.5% acetic acid was used as the mobile phase in isocratic mode at a flow rate of 1 mL per minute. The injection volume was 20 μL and the UV detector was set at 254 nm.

Urine creatinine levels were measured to normalize the contaminant concentration to present the chemical compounds of samples in terms of urinary metabolites per creatinine (µg/g cr).

### Quality control

The limit of detection (LOD) and limit of quantification (LOQ), determined as the concentrations equivalent to three and ten times the noise of the quantifier ion for a blank sample, were considered the quality control method. The LOD and LOQ for urinary metabolites of BTEX are shown in Table 1.

**Table 1:** Limit of detection (LOD) and limit of quantification (LOQ) for urinary metabolites of BTEX compounds (t,t MA, HA, MHA, MA, PGA).

Metabolite	LOD (µg/mL)	LOQ (µg/mL)
Trans, trans-muconic acid (t,t MA)	0.07	0.23
Hippuric acid (HA),	5.1	16.72
Methyl hippuric acid (MHA)	6	20
Mandelic acid (MA)	5.5	18.3
Phenylglyoxylic acid (PGA)	0.50	1.68

### Statistical approach

Descriptive analysis was conducted using frequency, frequency percentage, mean and standard deviation. Before any statistical test, the data normality test was performed using the Kolmogorov–Smirnov test to determine the type of statistical test. Due to the Non-normal distribution of data, a one-sample sign test was used to compare the mean concentration of urinary metabolites with the threshold limit value (TLV). The concentration of urinary biomarkers in the control and case groups and the concentration comparison before and after the shift was performed using the Mann–Whitney U test. A significant level in these analyses was less than 0.05 (p < 0.05). Finally, the logistic regression method was used for regression modeling. After examining all demographic and other variables in the exposed and control group, including smoking status, proximity to possible sources emissions of BTEX, heating devices, etc., the variables were entered into the model with a significant level of 0.25. Data analysis was performed using SPSS software version 21 (SPSS Inc. Chicago, IL).

### Ethics approval

Shiraz University of Medical Sciences Ethics Committee approved the study protocol by the code of “IR.SUMS.REC.1398.1267”.

### Consent to participate

The informed consent form was received from each subject.

## Results

### Personal characteristics of the participants

The results related to demographic characteristics and other general parameters of the workers in the case and control groups are shown in Table 2. In the present study, 112 urine samples were collected from WWTP employees. Twenty-six workers were in the administrative, and 30 were in the operational parts of the WWTP. All participants except three of the administrative employees (control group) were male with a mean age of 41.15 ± 0.28 years and a body mass index (BMI) of 23.99 ± 9.57. Fifteen workers in the case group and 11 in the control group were smokers.

**Table 2:** Socio-demographic characteristics of the studied population.

Variables	Control group	Exposed group
n	26	30
Age (years)	43.88 (8.15)^a^	38.62(7.17)^a^
Body mass index (kg/m^2^)	26.50 (3.31)^a^	26.20 (8.03)^a^
Normal weight (18.5–25)	7 (26.9%)	13 (43.3%)
Overweight (> 25)	15 (57.7%)	11 (36.7%)
Obesity(30–39.9)	3 (11.5%)	2 (6.7%)
**Education level**		
Less than a diploma	16 (61.5%)	8 (26.7%)
Diploma	1 (3.8%)	4 (13.3%)
Bachelor		9 (30%)
Masters and more		2 (6.7%)
Average working hours	7.94 (1.02)^a^	9.36 (4)^a^
**Smoking or exposure to secondhand smoke (home or/and work)**		
Yes	7 (26.9%)	15 (50%)
No	19 (73.1%)	15 (50%)
**Traffic situation**		
Mild	16 (61.5%)	15 (50%)
Medium	9 (34.6%)	14 (46.7%)
Heavy	1 (3.8%)	1 (3.3%)
**Heating devices**		
With flame	7 (26.9%)	23 (76.7%)
No flame	19 (73.1%)	7 (23.3%)
**Proximity to emission sources**		
Yes	13 (50%)	17 (56.7%)
No	13 (50%)	13 (43.3%)

^a^Arithmetic mean (standard deviation).

There was no significant difference between the exposed and control groups for age, weight, height, and BMI (p > 0.05). As indicated by the data given by the participants, three workers in the control group and five in the exposed group were exposed to cigarette and tobacco smoke in the last 48 h. Four workers in the control group kept solvents such as kerosene, gasoline, thinner, or other solvents at home during the previous 24 h. The control group spent 14.4 ± 16.16 h at home and 7.80 ± 2.81 h at work. The exposed group spent an average of 13.57 ± 3.98 h at home and 9.69 ± 3.48 h at work over the past 24 h.

### Urinary concentrations of BTEX in exposed and control groups

**Figure 1:**
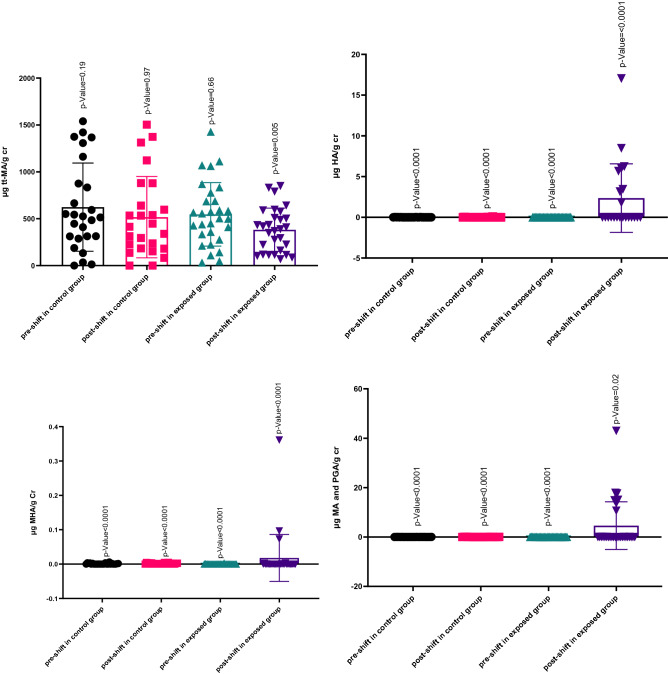
Scatter plot diagram of mean and SD for tt-MA, HA, MHA, and MA and PGA with the TLV.

Statistical results related to BTEX metabolites in the urine concentrations of the exposed and control groups are presented in Table 3. The mean concentration of urinary BTEX metabolites ranged from 546.43 (μg/g cr) for tt-MA to 0.006 (μg/g cr) for MHA acid. The mean and SD of concentration of urinary BTEX metabolites in the exposed and control groups are shown in Fig. 1.

**Table 3:** Descriptive statistics of urinary metabolites biomonitoring to BTEX (μg/g cr) before and after shiftwork in exposed and control groups.

Compounds	Pre-shift in exposed (μg/g cr)	Post-shift in exposed (μg/g cr)	Pre-shift in control (μg/g cr)	Post-shift in exposed (μg/g cr)	Comparison pre-shifts in two groups	Comparison post-shifts in two groups
	Mean ± SD	Mean ± SD	Mean ± SD	Mean ± SD	p-value	p-value
tt-MA	546.43 ± 338.90	381.94 ± 231.21	623.28 ± 470.30	516.74 ± 433.74	0.08	0.009
HA	0.006 ± 0.006	2.36 ± 4.21	0.006 ± 0.009	0.01 ± 0.02	0.04	< 0.0001
MHA	0.0002 ± 0.0006	0.01 ± 0.06	0.01 ± 0.01	0.01 ± 0.01	< 0.0001	0.01
MA and PGA	0.02 ± 0.09	17.67 ± 14.85	0.02 ± 0.009	0.02 ± 0.006	0.90	< 0.0001

The results of the statistical differences test comparing the metabolites of BTEX compounds in the exposed and control groups before and after work shifts are shown in Table 3. These results indicate a significant difference in the concentrations of tt-MA, HA, MHA, and MA and PGA after the end of the shift in the exposed and control groups (p < 0.05). The difference between the concentration of HA and MHA before the start of the shift in the exposed and control groups was statistically significant (p < 0.05). For comparison reasons, there was no significant difference in creatinine concentration before and after the work shift in the control group (p > 0.05).

The difference between the concentrations of urinary BTEX metabolites in each study group is presented in Table 4. This study showed a significant difference between tt-MA acid, Hippuric, Mandelic, and PGA before and after the end of the shift in the exposed group (p < 0.05). In addition, tt-MA, and creatinine before and after work shifts in the case group showed a significant difference (p < 0.05).

**Table 4:** Difference between urinary metabolites biomonitoring to BTEX (μg/g cr) before and after work shift in exposed and control groups.

Compounds	Control group p-value	Exposed group p-value
tt-MA	0.01	0.04
HA	0.20	0.01
MHA	0.47	0.16
MA and PGA	0.39	0.01
Creatinine	0.002	0.52

We are assessing the concentration of urinary metabolites of BTEX in the exposed and control groups before and after the work shift with the TLV in urine, as shown in Table 5 and Fig. 1. Most of the studied compounds showed a higher statistically significant concentration than the standard values.

**Table 5:** Comparison urinary metabolites of BTEX compounds (μg/g cr) before and after shiftwork in exposed and control groups with TLV.

Compounds	TLV	Pre-shifts in controls p-value	Post-shifts in controls p-value	Pre-shifts in exposed p-value	Post-shifts in exposed p-value
tt-MA	500 (µg/g cr)	0.19	0.97	0.66	0.005
HA	1.6 (g/g cr)	< 0.0001	< 0.0001	< 0.0001	< 0.0001
MHA	1.5 (g/g cr)	< 0.0001	< 0.0001	< 0.0001	< 0.0001
MA and PGA	0.15 (g/g cr)	< 0.0001	< 0.0001	< 0.0001	< 0.0001

Results of the correlation between urinary BTEX metabolites in the exposed and control groups (before and after work shift) showed only a significant relationship between measured tt-MA (p = 0.04, r = 0.37) and MA and PGA (p = 0.01, r = 0.43) before the shift in the group exposed to HA measured before the shift in the same group.

### Logistic regression analysis

For regression modeling, all variables were examined (including age, cigarette and tobacco smoke, proximity to emission sources, creatinine, the heating device used by participants, and traffic situation), considering the significance level of 0.25. Individual variables for each exposure group and control group constitute age, cigarette and tobacco smoke, a heating device (including flammable and without flame), and creatinine concentration entered into backward logistic regression models: conditional with p-values of 0.02, 0.08, 0.001, and 0.02, respectively.

After adjusting the age, the absolute values of the concentration of metabolites in the control group were 14% higher than in the exposed group. Adjusting for the difference in creatinine concentration before and after the work shift, metabolites’ concentration was 0.03% more in the exposed group than the control group. Finally, the heating device affected the concentration of pollutants in the exposed groups compared to the control 23.85 times. Univariate and multivariate logistic regression of exposed and control groups with all existing variables has been shown in Table 6 (by the detail of crude and adjusted OR).

**Table 6:** Univariate and multivariate logistic regression on the concentration of the urinary metabolite of BTEX in exposed and control groups.

Variable	Exposed and Control group in crude				Exposed and Control group after adjusting			
	p-value	Crude OR	95% CI for OR		p-value	OR adjusted	95% CI for OR	
			Lower	Upper			Lower	Upper
Age	0.023	0.91	0.84	0.98	0.015	0.856	0.755	0.971
Smoking	0.22	2.38	0.59	9.64	0.32	2.37	0.42	13.31
Proximity to sources of emissions	0.59	1.15	0.61	1.69	–	–	–	–
Heating device	< 0.001	13.01	3.11	54.32	0.001	23.858	3.644	156.210
Traffic situation near the participants home	0.96	0.93	0.85	1.01	–	–	–	–
Concentration of creatinine	0.009	1.03	1.007	1.05	0.003	1.039	1.013	1.066

## Discussion

Volatile Organic Compounds (VOCs) are plentiful in industrial and municipal wastewater^7^. The main sources of VOCs in wastewater are water supply, industrial and commercial processes, household and consumer goods, surface runoff, and chemical and biological reactions during water and wastewater treatment^35^. In WWTPs, VOCs are mainly delivered to the air through dispersion and volatilization^35^. Most research on VOCs in WWTPs has been focused on processes that control or remove VOCs from wastewater^36,37^. Considering the presence of BTEX and its by-products in WWTPs in the literature^38,39^, and recognizing benzene and toluene that were released from different treatment units of this WWTP^33^, the present study aims at assessing the exposure of workers in the WWTP to BTEX compounds (benzene, toluene, ethylbenzene, xylene), as the first biomonitoring campaign using metabolites of BTEX in the urine ever accomplished in the WWTPs of Middle East.

The results of this study demonstrated a high concentration of urinary metabolites of BTEX compounds in both exposed and control groups. Among the studied variables in this research (smoking, using heating devices, age, and creatinine concentration), a significant relationship between age, use of the flame heating device, and creatinine concentration by changing the concentration of benzene, toluene Ethylbenzene, meta, para-xylene metabolites was presented in the urine of study participants.

Based on the results of this study, no significant difference was observed between the exposed and control groups in terms of age, weight, height, and BMI. The current results were consistent with those of the study of Rafiei et al. (2018) and Moridzadeh et al. (2020)^40,41^.

We report that the detection level of different BTEX metabolites was different due to variations in the absorption, metabolism, and distribution of these compounds^42^. Our study results showed that the control group was exposed to higher concentrations of BTEX, contrasted with workers in past investigations in various occupational and environmental exposures^9,41,43,44^.

The highest and lowest urinary metabolites of BTEX compounds were observed in the group exposed, resulting exposed to benzene and ethylbenzene, respectively. These results are in agreement with the study by Oh et al., in which the highest concentration of urinary metabolites among smokers and non-smokers housekeepers in North Korea with a geometric mean and standard error (SE) for all studied participants was 46.80 ± 2.48 (μg/g cr) belonging to tt-MA^43^. A range of benzene levels from the least of 0.006 ppm to the most elevated of 0.25 ppm was correlated with the average tt-MA metabolite as 360 ± 340 µg/g Cr, representing 83.6% of benzene exposure^45^. The fluctuation in urinary tt-MA levels between workers could be clarified by various competitive paths of benzene metabolism for muconic acid and other metabolites requiring GSTT (glutathione/sulfotransferase) activity. In addition, the major metabolic stage, CYP2E1 (cytochrome P4502E1), may be reduced to ttMA metabolites by alcohol intake^46^.

More than half of the measured metabolites were more than the TLV of these compounds in the urine. Even though the control group's exposure level in the workplace was not very high, in some cases close to zero, high levels of tt-MA were detected in all biological samples. It confirms that benzene can be considered one of the widespread environmental pollutants^47^ and that there are many sources for urinary metabolites of benzene^47,48^.

Intergroup study of urinary metabolite levels after shift work in control and exposed groups showed a significant difference for all metabolites (p < 0.05). In the present study, the average urinary level of tt-MA measured in the samples of workers after the work shift was statistically different from the samples before the shift. Still, the average level of these metabolites in the group of office workers (control) before and after work shift only showed a significant difference for urinary tt-MA level. In contrast, no significant difference was observed in the other three groups of urinary metabolites in the control group (p > 0.05). A significant amount of tt-MA in the morning sample of workers might be due to the internal distribution time of benzene with tissues (e.g., adipose tissue). It indicates that a large amount of benzene may be accumulated depending on the worker's living conditions. Then redistribution, metabolism, and excretion can occur in the form of tt-MA. Transdermal exposure to benzene can be another important source of delay in the presence of tt-MA in urine^47^.

Biomonitoring of occupational benzene exposure tends to be distorted by factors affecting tt-MA level, including consuming food of sorbic acid, exposure to the external environment, individual susceptibility, and work behavior^49^. However, something that should be taken into account is that nowadays, foods containing preservatives are an essential part of the nutrition and diet of most people in society and will have a notable effect on urine tt-MA. Therefore, tt-MA cannot be a specific and highly sensitive biomarker for illustrating benzene exposure. However, the levels of other urinary metabolites in the exposed group were increased in concentration/gr of creatinine after their work shift.

Contrary to the results of previous investigations, the urinary level of BTEX metabolites of WWTP workers in the analysis of logistic regression was not significantly associated with smoking. Benzene and other BTEX compounds are components of ambient tobacco smoke, and in the results of a study by Jalai et al. 2017 the urinary tt-MA level in the morning and evening for smokers and non-smokers of police officers was reported to be 571 ± 120, 820 ± 187, 321 ± 145, and 544 ± 160 (mg/g creatinine), respectively^47^. This concentration for urban residents was 425 ± 198, 501 ± 161, 224 ± 55, and 275 ± 57 (mg/g cr), respectively^47^, which is consistent with the results of our study. The tt-MA levels in the urine of chemical company workers were measured at 1824 ± 147 (mg/g cr) and 4616 ± 225 (mg/g cr) in the morning and evening of smokers and 987 ± 111 (mg/g cr) and 2271 ± 780 (mg/g cr) in the morning and evening of non-smokers^47^ which is more than twice the measured tt-MA concentration in the urine samples of WWTP employees in our study. However, the results of rural residents' tt-MA were relatively lower than the concentrations of tt-MA showed in our study^47^. Contrary to our study, the urinary tt-MA concentrations in all these population groups were almost twice higher in the smokers than the non-smokers.

The heating device use and urinary creatinine levels showed a positive correlation in the exposed group, whereas age caused a decrease in the concentration of these compounds in this group. The results of the study by Wheeler et al.^50^ showed that benzene concentration predictors in indoor places were considered frequent smoking at home, the property's garage, remodeling activities in the past 90 days, opening of windows in the previous week to let fresh air into the house, and utilization of candles during the last week. The predictors of ethylbenzene, *m*-, *p*-xylenes: the existence of a garage on the property, renovated last month, utilization of perfume on the previous day, and utilization of paint remover the week before. In addition, an additional predictor for *m*-, *p*-xylenes was the habitude of household smoking. The toluene predictive indicators were home garages, newer homes, painting or using paint remover in the last week, and frequent home smoking. The type of heating source in the house was not a predictor for BTEX concentration in the Wheeler et al. study^50^. Canada's long-term residential indoor air quality health guidelines for toluene^51^ (2300 μg/m^3^) are within the range of values established by other recognized international organizations (300 to 5000 μg/m^3^) ^52–55^.

The strength of our study is assessing the exposure to BTEX, using metabolized biomarkers in the WWTP workplace for the first time in the Middle Eastern. Some studies have evaluated the amount of BTEX emissions by directly measuring the concentration of internal BTEX in other occupations^56–58^. In all studies, despite the adjustment for many essential factors such as BMI, age, etc., there is a possibility of complications in unknown factors and possible sources. However, the biological monitoring method integrates all exposure pathways and can include random and unexpected exposures in the exposure measurement results^59^. Biological monitoring of BTEX urinary metabolites in populations exposed to these volatile pollutants in the air provides valuable information to health authorities about its environmental exposure limit reference and, like an easy-to-access biomatrix, provides an accurate assessment of exposure to these compounds through the air^60,61^.

However, the limitations of our investigation center around the relatively small sample size in some participant groups (smokers or exposed to environmental emissions). However, this was the most significant number of samples possibly accessible at the studied center (WWTP). In addition, the limitations of the method concern sampling; since spotted urine samples represent only recent exposures, the results of this study cannot be used to interpret long-term BTEX exposure in workers, and these results should be interpreted carefully to be confirmed by other studies.

Finally, the lack of specific questions about personal protective equipment, indoor ventilation, or seasonal and daily changes was a limitation of our investigation. Other epidemiological and experimental studies have shown that continuous ventilation reduces the degree of internal toxins^62,63^. Safety measures may be considered an essential determinant of BTEX occupational exposure^40,64,65^. Besides, several studies have suggested that tt-MA may not be a reliable biomarker for exposure to low levels of benzene. However, it is still considered a useful biomarker for assessing benzene metabolites in urine with cost-effective analytical screening purposes^47,66^. However, smoking and alcohol intake should be controlled as confounding factors in selecting specific metabolites as benzene exposure biomarkers. Eating of preserved food sources containing sorbic acid could be additionally limited for the use of specific tt-MA in biological monitoring of exposure assessment to benzene in workplaces, according to the previous suggestion. Last but not least, this study examined the estimation of the potential exposure through the skin, food, or inhalation contact only through a questionnaire. Therefore, future studies can investigate the emission sources of BTEX for WWTP workers.

## Conclusions

This investigation attempted to evaluate the exposure of WWTP workers to BTEX compounds. Our findings showed that WWTP workers were exposed to BTEX compounds in some cases more than the permitted reference values, especially for benzene. Therefore, WWTPs should be considered as a source of BTEX, and employees working in such facilities may potentially be exposed to these volatile compounds.

The results of the present study indicated that age, use of flame heaters, and creatinine concentration are significant predictors of increasing concentrations of urinary BTEX metabolites. Therefore, it tends to be proposed that protective methodologies, like functional chemical masks, e.g., VOC absorbing masks, can be utilized to decrease worker exposures. It is also suggested that it is important to implement periodic health checks for WWTP workers.

To the best of the authors’ knowledge, the present study is the first study with biological monitoring approach to assess the WWTP workers' exposure to BTEX, aiming to create fitting programs to promote occupational health. As the monitoring of BTEX compounds in ambient air was beyond our investigation's scope, future research is suggested to assess the relative contribution of various BTEX exposure pathways in these environments.

## Data Availability

The data that support the findings of this study are available from the corresponding author, [M.A], upon reasonable request.
